# Medicaid expansion and inpatient hospital charges among women with major depressive disorders

**DOI:** 10.1371/journal.pone.0335006

**Published:** 2026-06-25

**Authors:** Oluwasegun Akinyemi, Mojisola Fasokun, Fadeke Ogunyankin, Gabriella Kuffour, Samar K. Khalil, Ofure Omokhodion, Rachael Oyebade, Chioma Ekwunazu, Ayomide Ogunsakin, Miriam Michael, Guoyang Luo

**Affiliations:** 1 The Clive O Callender Outcomes Research Center, Howard University College of Medicine, Washington District of Columbia, United States of America; 2 Department of Internal Medicine, University Hospitals Cleveland Medical Center, Cleveland, Ohio, United States of America; 3 Department of Research Data Science and Analytics, Cook Children’s Health Care System: Cook Children’s Medical Center, Fort Worth, Texas, United States of America; 4 Department of Obstetrics and Gynecology, University of Central Florida, Orlando, Florida, United States of America; 5 Department of Family Medicine, University of Iowa Hospital and Clinics, Iowa City, Iowa, United States of America; 6 Department of Internal Medicine, Howard University College of Medicine, Washington District of Columbia, United States of America; 7 Department of Obstetrics and Gynecology, Inova Health System, Fairfax, Virginia United States of America; Emory University, UNITED STATES OF AMERICA

## Abstract

**Objective:**

To examine the association between Medicaid expansion under the Affordable Care Act (ACA) and total inpatient hospital charges among women hospitalized with major depressive disorder (MDD), comparing Maryland and New Jersey, two Medicaid expansion states with distinct payment systems, to Florida, a non-expansion state.

**Methods:**

We conducted a retrospective cohort study using data from the State Inpatient Databases for Maryland, New Jersey, and Florida from 2007 to 2020. The study population included women aged 18–64 years admitted with a primary diagnosis of MDD. The pre-ACA period was defined as 2007–2013, and the post-ACA period as 2014–2020. Difference-in-differences (DID) models with robust standard errors were used to estimate changes in total inpatient hospital charges, adjusting for demographic, socioeconomic, and clinical characteristics. Stratified analyses by race/ethnicity and insurance type were conducted to assess heterogeneity in policy effects.

**Results:**

In adjusted analyses, Medicaid expansion was associated with divergent changes in hospital charges across expansion states. Compared with Florida, Maryland experienced a relative post-ACA reduction (DID estimate − $2,313; 95% CI: − $2,549 to −$2,078). In contrast, New Jersey had a post-ACA increase (+$3,366; 95% CI: $2,746 to $3,987). In Maryland, post-ACA reductions were observed across all racial and ethnic groups and insurance categories, with the largest decreases among uninsured and Medicaid-covered patients. New Jersey demonstrated heterogeneous patterns, including charge increases across most payer groups.

**Conclusion:**

The effects of Medicaid expansion on inpatient charges among women with MDD differed across states, reflecting variations in payment regulation and hospital pricing. Maryland’s reductions suggest that coupling coverage expansion with cost‑containment mechanisms may help constrain hospital charge growth, with potential implications for the financial burden of mental health care.

## Introduction

Major depressive disorder (MDD) is one of the leading causes of disability worldwide and remains a major contributor to health care utilization and costs in the United States [1]. Recent estimates suggest that over 21 million U.S. adults experience at least one major depressive episode annually, with women nearly twice as likely as men to be affected [2,3]. Moreover, the economic burden of MDD exceeds $320 billion each year, driven largely by healthcare expenditures and productivity losses [4]. This burden is particularly pronounced among women of reproductive age, given their higher prevalence of MDD and frequent interactions with the healthcare system [5–9]. Beyond its profound individual and societal burden, inpatient psychiatric care accounts for much of MDD-related spending, and rising hospital charges have heightened concerns about affordability and equitable access [4,10,11]. Addressing the financial burden of psychiatric care [12,13] and reducing disparities in access to essential services [14] are therefore pressing public health priorities.

The passage of the Patient Protection and Affordable Care Act (ACA) in 2010 represented a landmark reform in U.S. health care, with provisions aimed at expanding insurance coverage, reducing uncompensated care, and improving access to essential services [15]. Among its most transformative elements was Medicaid expansion, which extended eligibility to millions of low-income adults [16]. Prior research has shown that Medicaid expansion is associated with improved insurance continuity, greater access to mental health services, and reductions in unmet mental health needs [17–19]. These changes are particularly consequential for women with chronic mental health conditions such as MDD, for whom financial barriers frequently delay or limit access to care [20,21].

Despite these documented coverage gains, the extent to which Medicaid expansion translates into lower inpatient hospital charges remains less well understood. States differ substantially in hospital payment regulation, pricing flexibility, and market dynamics, all of which may influence how insurance expansion affects hospital charges [22,23]. As a result, the financial impact of Medicaid expansion may vary depending on the broader policy and payment environment in which it is implemented [24,25].

Maryland, New Jersey, and Florida provide a useful policy contrast for examining these dynamics. Both Maryland and New Jersey implemented Medicaid expansion in 2014; however, Maryland operates under an All-Payer Hospital Payment System that regulates hospital payments across payers, potentially limiting price growth and reducing cost shifting [25–27]. In contrast, although New Jersey expanded Medicaid during the same period, it relies on more traditional hospital payment structures without an all-payer model. Florida, meanwhile, did not adopt Medicaid expansion and continues to operate under conventional hospital payment mechanisms [28]. This variation in expansion status and payment models creates a natural policy experiment to evaluate how Medicaid expansion within differing reimbursement environments is associated with changes in inpatient hospital charges among women hospitalized with MDD.

The objective of this study was to evaluate whether Medicaid expansion under the ACA is associated with changes in inpatient hospital charges among women hospitalized with MDD, using a difference-in-differences (DID) framework. By examining outcomes across states with distinct policy and payment contexts, this study contributes new evidence at the intersection of health policy, women’s mental health, and hospital financing, with implications for financial protection and equity in psychiatric care.

## Methods

### Study design and data source

We conducted a retrospective; multi-state DID study to evaluate the association between Medicaid expansion and inpatient hospital charges among women hospitalized with MDD. Data were obtained from the Healthcare Cost and Utilization Project (HCUP) State Inpatient Databases, which capture all inpatient discharges from participating hospitals and provide a comprehensive representation of hospitalizations within each state [26–28]. The analytic period spanned January 1, 2007, through December 31, 2020.

Three states were included in the analysis. Maryland and New Jersey were selected as Medicaid expansion states, both implementing expansion in January 2014, while Florida served as a non-expansion comparison state. Maryland operates under an All-Payer Hospital Payment System that regulates hospital payments across payers, whereas New Jersey and Florida use traditional hospital payment structures. Including New Jersey allowed us to assess whether changes in hospital charges associated with Medicaid expansion differed across expansion states with distinct payment environments. This study was conducted in accordance with the Strengthening the Reporting of Observational Studies in Epidemiology (STROBE) reporting guidelines to promote transparent and comprehensive reporting of observational research.

### Study population

The study population included women aged 18–64 years hospitalized with a primary or secondary diagnosis of major depressive disorder, identified using International Classification of Diseases, Ninth and Tenth Revision (ICD-9 and ICD-10) diagnosis codes. Major depressive disorder was identified using International Classification of Diseases, Ninth and Tenth Revision (ICD-9 and ICD-10) diagnosis codes (S1 Table). The final case definition was restricted to ICD-9 and ICD-10 codes corresponding to established severity-based subcategories of major depressive disorder within the F32 and F33 families. ICD-10 code F33.8 (Other recurrent depressive disorders) was excluded because it captures heterogeneous atypical presentations that do not correspond to a specific severity-based subcategory of MDD and lacks a direct ICD-9 equivalent, which would introduce coding asymmetry across the ICD-9 to ICD-10 transition period. ICD-10 code F39 (Unspecified mood disorder) and its ICD-9 equivalent (296.90) were similarly excluded to improve diagnostic specificity, as this code encompasses mood disturbances not restricted to major depressive disorder and could introduce misclassification bias [29,30].Women aged 65 years and older were excluded because of near-universal Medicare eligibility, which could attenuate or confound the effects of Medicaid expansion. All hospitalizations meeting these inclusion criteria were retained for analysis, and no sampling was performed.

### Outcome measure

The primary outcome was total inpatient hospital charges (TOTCHG), a continuous measure available in the HCUP SID that reflects facility-level charges for each hospitalization [31]. TOTCHG represents cleaned and edited total charges following HCUP’s standardized quality-control procedures, including rounding to the nearest dollar and recoding of implausible values based on year-specific thresholds. Because hospital charge data are typically right-skewed, we examined the distribution of TOTCHG for extreme values. No additional trimming or winsorization was performed, as HCUP data-cleaning procedures are designed to identify and correct implausible charge amounts. While TOTCHG does not represent actual payments, reimbursements, or patient out-of-pocket costs, it provides a consistent and widely used measure of hospital pricing behavior associated with inpatient care across states and over time. Charges were analyzed in nominal dollars and were not adjusted for inflation; the difference-in-differences design partially addresses this by absorbing secular price trends common to all states into the time-period effects.

### Independent variables

The primary exposure of interest was Medicaid expansion status. States were categorized as expansion states (Maryland and New Jersey) or non-expansion states (Florida). Time was categorized into pre-ACA and post-ACA periods. The pre-ACA period was defined as 2007–2013, and the post-ACA period was defined as 2014–2020, corresponding to the implementation of Medicaid expansion in the included expansion states.

### Covariates

Covariates were selected a priori based on prior literature and conceptual relevance as potential confounders of the association between Medicaid expansion and inpatient hospital charges. We adjusted for patient demographic, socioeconomic, and clinical characteristics known to influence healthcare utilization and costs.

Demographic variables included age (modeled continuously) and race/ethnicity, categorized as White, Black, Hispanic, and Other. Socioeconomic status was measured using median household income quartiles based on patient ZIP code. Primary expected payer was classified as Medicaid, Medicare, private insurance, uninsured, or other/no charge. Clinical comorbidities included hypertension, diabetes, obesity, and smoking status. Management of MDD was categorized as medical management, psychotherapy, and electroconvulsive therapy.

### Statistical analysis

Descriptive statistics were used to summarize patient and hospitalization characteristics by state and ACA period. Baseline characteristics were reported as counts and percentages for categorical variables and as means with standard deviations for continuous variables. Categorical variables were compared across the three groups using chi-square tests, while continuous variables were analyzed using one-way analysis of variance (ANOVA) or the Kruskal–Wallis test, as appropriate.

We then estimated DID models using weighted linear regression with robust standard errors. The primary model included indicators for Medicaid expansion status, ACA period, and their interaction. The interaction term between expansion status and post-ACA period provided the DID estimate of the association between Medicaid expansion and changes in inpatient hospital charges. To assess the parallel trends assumption underlying the difference-in-differences design, we examined pre-expansion trends in mean log inpatient hospital charges across states (S1 Fig). All analyses were conducted using Stata version 16.0 (StataCorp LLC, College Station, TX). Statistical significance was defined as a two-sided P value <0.05.

### Missing data

We evaluated the extent of missing data across study variables. Missingness was minimal (< 5% for all covariates). Hospitalizations with missing outcome data were excluded, and complete-case analysis was used for covariates because the proportion of missing observations was small and unlikely to meaningfully bias estimates.

### Stratified and sensitivity analyses

To examine potential heterogeneity in policy effects, we conducted stratified analyses by race/ethnicity and primary expected payer. In addition, sensitivity analyses incorporated three-way interaction terms among Medicaid expansion status, ACA period, and insurance type or race/ethnicity. These models allowed us to assess whether changes in hospital charges were concentrated among populations most directly affected by Medicaid expansion and to explore differences in expansion effects across states with distinct payment environments.

### Ethical considerations

This study used de-identified HCUP SID data and was not considered human subjects research. Institutional Review Board approval and informed consent were therefore not required.

## Results

Table 1 summarizes the baseline demographic and clinical characteristics of women hospitalized with MDD across Florida, Maryland, and New Jersey before and after implementation of the Medicaid expansion under the ACA. The study included 345,494 hospitalizations, of which 143,236 occurred in the pre-ACA period and 202,258 in the post-ACA period.

**Table 1:** Baseline Demographic and Clinical Characteristics of Patients in Florida, Maryland and New Jersey Pre- and Post-ACA Implementation.

Variables (N = 345,494)	Pre-ACA (n = 143,236)				Post-ACA (n = 202,258)			
	Florida 88,801 (62.0%)	Maryland 26,364 (18.4%)	New Jersey, 28,071 (19.6%)	P-value	Florida 136,448 (67.5%)	Maryland 29,795 (14.7%)	New Jersey 36,015 (17.8%)	p
**Age, year, Mean ± SD**	42.0 ± 12.8	40.4 ± 12.5	41.0 ± 12.8	<0.01	39.8 ± 14.0	37.8 ± 13.6	39.0 ± 13.8	<0.01
**Total Charges**	12635.6 ± 13,204.7	7,811.9 ± 10,566.9	33,100.8 ± 36,519	<0.01	16,541.4 ± 18,123	24,101 ± 26,054	39,774.4 ± 42,418.6	<0.01
**Race/Ethnicity**				< 0.01				< 0.01
White	63,288 (72.0%)	15,664 (60.1%)	17,648 (63.9%)		88,820 (66.1%)	16,652 (57.6%)	21,294 (60.5%)	
Black	10,444 (11.9%)	8,521 (32.7%)	4,457 (16.1%)		20,564 (15.3%)	9,342 (32.3%)	5,882 (16.7%)	
Hispanic	11,501 (13.1%)	708 (2.7%)	3,826 (11.3%)		21,476 (15.9%)	1,510 (5.2%)	5,126 (14.5%)	
Other	2, 664 (3.0%)	1,159 (4.4%)	1,682 (6.1%)		3,468 (2.6%)	1,408 (4.9%)	2,886 (8.2%)	
**Income by Zip Code**				<0.01				< 0.01
Lower-Income	53,446 (62.0%)	8,192 (31.7%)	7,632 (27.5%)		94,544 (72.3%)	7,867 (26.8%)	9,728 (27.2%)	
Higher-Income	32,681 (37.9%)	17,647 (68.3%)	20,149 (72.5%)		36,216 (27.7%)	21,472 (73.2%)	26,031 (72.8%)	
**Primary Insurance**				<0.01				<0.01
Medicare	17,004 (19.1%)	3,141 (11.9%)	3,290 (11.7%)		22,217 (16.2%)	3,488 (11.7%)	3,730 (10.4%)	
Medicaid	16,128 (18.2%)	8,617 (32.7%)	5,247 (18.7%)		32,551 (23.9%)	13,340 (44.8%)	15,617 (43.4%)	
Private Insurance	37,230 (41.9%)	10,461 (39.7%)	10,744 (38.3%)		49,895 (36.6%)	10,878 (36.6%)	12,552 (34.9%)	
Un-insured	11,384 (12.8%)	3,303 (12.5%)	7,480 (26.6%)		19,519 (14.3%)	759 (2.5%)	1,961 (5.4%)	
Other	7,055 (7.9%)	810 (3.1%)	1,310 (4.7%)		12,266 (9.0%)	1,281 (4.3%)	2,147 (5.9%)	
**Hypertension**	22,540 (25.4%)	7,551 (28.6%)	6,661 (23.7%)		30,991 (22.7%)	7,314 (24.5%)	7,492 (20.8%)	
**Obesity**	7,915 (8.9%)	3,784 (14.4%)	2,072 (7.4%)	< 0.01				< 0.01
**Diabetes Mellitus**	8,895 (10.0%)	3,348 (12.7%)	2,732 (9.7%)	<0.01	9,162 (6.7%)	3,075 (10.3%)	2,086 (5.8%)	<0.01
**Smoking**				<0.01				<0.01
Never	72,577 (81.7%)	18,418 (69.9%)	21,898 (78.0%)		98,763 (72.4%)	18,933 (63.5%)	24,335 (67.6%)	
Former	923 (1.0%)	413 (1.6%)	464 (1.6%)		3,440 (2.5%)	1,290 (4.3%)	1,405 (3.9%)	
Current	15,301 (17.2%)	7,533 (28.6%)	5,709 (20.3%)		34,245 (25.1%)	9,572 (32.1%)	10,275 (28.5%)	
**Management of MDD**				<0.01				<0.01
Medication	86,705 (97.6%)	25,583 (97.0%)	26,338 (93.8%)		135,248 (99.1%)	29,152 (97.8%)	35,161 (97.6%)	
Psychotherapy	503 (0.6%)	215 (0.8%)	1,058 (3.8%)		888 (0.7%)	497 (1.7%)	676 (1.9%)	
ECT	1,593 (1.8%)	566 (2.2%)	675 (2.4%)		312 (0.2%)	146 (0.5%)	178 (0.5%)	

Significant differences were observed across states for most characteristics in both periods (Table 1). Florida accounted for the largest proportion of hospitalizations pre-ACA (62.0%) and post-ACA (67.5%), followed by New Jersey (19.6% and 17.8%, respectively) and Maryland (18.4% and 14.7%, respectively). Mean age declined modestly following ACA implementation across all states (Florida: 42.0 ± 12.8 vs 39.8 ± 14.0 years; Maryland: 40.4 ± 12.5 vs 37.8 ± 13.6 years; New Jersey: 41.0 ± 12.8 vs 39.0 ± 13.8 years; P < 0.01 for all comparisons).

Total hospital charges differed substantially by state (Table 1). Pre-ACA, mean charges were highest in New Jersey ($33,100.8 ± 36,519.0), followed by Florida ($12,635.6 ± 13,204.7), and lowest in Maryland ($7,811.9 ± 10,566.9). Post-ACA, charges increased across all states, remaining highest in New Jersey ($39,774.4 ± 42,418.6), followed by Maryland (~$24,101), and Florida ($16,541.4 ± 18,123.0).

Racial and ethnic distributions varied significantly across states (Table 1). Florida had a higher proportion of White patients both pre- and post-ACA (72.0% and 66.1%), whereas Maryland had the highest proportion of Black patients (32.7% and 32.3%). Hispanic patients were more frequently represented in Florida (13.1% pre-ACA; 15.9% post-ACA) and New Jersey (11.3% pre-ACA; 14.5% post-ACA) compared with Maryland (2.7% pre-ACA; 5.2% post-ACA).

Socioeconomic differences were also evident. Patients from lower-income ZIP codes were disproportionately represented in Florida before and after the ACA (62.0% and 72.3%), whereas Maryland and New Jersey had higher proportions of patients from higher-income neighborhoods (Table 1).

Insurance coverage shifted significantly after ACA implementation (Table 1). Medicaid coverage increased in all states, particularly in Maryland (32.7% to 44.8%) and New Jersey (18.7% to 43.4%), while the proportion of uninsured patients declined markedly in Maryland (12.5% to 2.5%) and New Jersey (26.6% to 5.4%).

Clinical comorbidities differed across states. Hypertension was most prevalent in Maryland pre-ACA (28.6%) but declined post-ACA (24.5%). Similarly, the prevalence of diabetes decreased across states following the ACA (P < 0.01). Smoking patterns also varied, with higher proportions of current smokers in Maryland and New Jersey compared with Florida in both periods (Table 1).

Management of MDD was consistent across states, with medication representing the primary treatment modality pre-ACA (93.8%–97.6%) and post-ACA (97.6%–99.1%). Psychotherapy and electroconvulsive therapy were used far less frequently (Table 1).

### Association between medicaid expansion and total inpatient hospital charges

Table 2 presents the DID estimates of the association between Medicaid expansion and total inpatient hospital charges among women hospitalized with MDD. Overall, significant differences in hospital charges were observed by expansion status and ACA period. Compared with Florida, Maryland experienced a substantially lower total hospital charges (−$6,381; 95% CI, − $6,540 to −$6,222), whereas New Jersey exhibited markedly higher charges (+$21,326; 95% CI, $21,014 to $21,638). Across all states, the post-ACA period was associated with higher hospital charges relative to the pre-ACA period (+$4,947; 95% CI, $4,801 to $5,093).

**Table 2:** Difference-in-Differences Estimates of the Association Between Medicaid Expansion and Total Hospital Charges Among Women Hospitalized with Major Depressive Disorder, Comparing Maryland and New Jersey With Florida.

Comparison	Contrast ($)	Std. Error	t value	P value	95% CI
Maryland vs Florida (overall)	−6,381.30	81.07	−78.72	<0.001	−6,540.19 to −6,222.41
New Jersey vs Florida (overall)	+21,326.03	159.02	134.11	<0.001	+21,014.36 to +21,637.70
Post-ACA vs Pre-ACA	4,946.68	74.40	66.49	<0.001	4,800.85 to 5,092.50
Maryland × Post-ACA	−2,313.16	120.18	−19.25	<0.001	−2,548.71 to −2,077.61
New Jersey × Post-ACA	3,366.10	316.60	10.63	<0.001	2,745.57 to 3,986.63

Florida served as the non–Medicaid expansion reference state. Maryland implemented Medicaid expansion in 2014 and operates an all-payer hospital payment system, while New Jersey expanded Medicaid in 2014 without an all-payer model and was included as a sensitivity analysis. Estimates represent contrasts of predictive margins from multivariable linear regression models with robust standard errors, adjusted for age, race/ethnicity, median household income by ZIP code, primary expected payer, hypertension, diabetes, obesity, smoking status, and management of major depressive disorder (medical management, psychotherapy, and electroconvulsive therapy). Three-way interaction terms among Medicaid expansion status, ACA period, and race/ethnicity or insurance type were included to generate difference-in-difference-in-differences (DDD) estimates. The interaction term (Expansion × Post-ACA) represents the difference-in-differences estimator. Charges are reported in US dollars.

In DID analyses, Medicaid expansion in Maryland was associated with a significant relative reduction in hospital charges in the post-ACA period compared with Florida (−$2,313; 95% CI, − $2,549 to −$2,078). In contrast, New Jersey experienced a significant relative increase in post-ACA hospital charges compared with Florida (+$3,366; 95% CI, $2,746 to $3,987) (Table 2).

### Race-stratified differences in hospital charges

Table 3 shows race- and ethnicity-stratified DID estimates. Significant heterogeneity was observed across racial and ethnic groups (joint F-test P < 0.001). In Maryland, Medicaid expansion was associated with consistent and statistically significant reductions in hospital charges across all racial and ethnic categories. Post-ACA reductions were observed among White (−$2,030; 95% CI, − $2,342 to −$1,717), Black (−$2,726; 95% CI, − $3,192 to −$2,261), Hispanic (−$3,054; 95% CI, − $3,993 to −$2,116), and other race groups (−$3,902; 95% CI, − $5,131 to −$2,674).

**Table 3:** Race-Stratified DID Estimates of the Association Between Medicaid Expansion and Total Hospital Charges Among Women Hospitalized With MDD, Comparing Maryland and New Jersey With Florida.

Race/Ethnicity	State Comparison	Contrast ($)	Std. Error	t value	P value	95% CI
White	Maryland × Post-ACA	−2,029.73	159.56	−12.72	<0.001	−2,342.46 to −1,717.01
Black	Maryland × Post-ACA	−2,726.33	237.34	−11.49	<0.001	−3,191.50 to −2,261.16
Hispanic	Maryland × Post-ACA	−3,054.43	479.01	−6.38	<0.001	−3,993.28 to −2,115.58
Other race groups	Maryland × Post-ACA	−3,902.44	626.69	−6.23	<0.001	−5,130.73 to −2,674.16
White	New Jersey × Post-ACA	2,136.12	393.35	5.43	<0.001	1,365.17 to 2,907.08
Black	New Jersey × Post-ACA	2,282.90	754.73	3.02	0.002	803.66 to 3,762.15
Hispanic	New Jersey × Post-ACA	−865.91	724.64	−1.19	0.232	−2,286.18 to 554.35
Other race groups	New Jersey × Post-ACA	20,835.33	1,553.26	13.41	<0.001	17,790.99 to 23,879.68

Florida served as the non–Medicaid expansion reference state. Maryland implemented Medicaid expansion in 2014 and operates an all-payer hospital payment system, while New Jersey expanded Medicaid in 2014 without an all-payer model and was included as a sensitivity analysis. Estimates represent contrasts of predictive margins from multivariable linear regression models with robust standard errors, adjusted for age, race/ethnicity, median household income by ZIP code, primary expected payer, hypertension, diabetes, obesity, smoking status, and management of major depressive disorder (medical management, psychotherapy, and electroconvulsive therapy). Three-way interaction terms among Medicaid expansion status, ACA period, and race/ethnicity or insurance type were included to generate difference-in-difference-in-differences (DDD) estimates. The interaction term (Expansion × Post-ACA) represents the difference-in-differences estimator. Charges are reported in US dollars.

In contrast, New Jersey exhibited post-ACA increases in hospital charges among White (+$2,136; 95% CI, $1,365 to $2,907) and Black women (+$2,283; 95% CI, $804 to $3,762), no statistically significant change among Hispanic women, and a pronounced increase among women classified as other race groups (+$20,835; 95% CI, $17,791 to $23,880). These results indicate that the association between Medicaid expansion and inpatient hospital charges varies substantially by race/ethnicity and state context.

### Insurance-stratified differences in hospital charges

Table 4 presents insurance stratified DID estimates. Significant heterogeneity was observed across insurance categories (joint F-test P < 0.001). In Maryland, Medicaid expansion was associated with significant post-ACA reductions in hospital charges across all insurance groups. The largest reductions were observed among uninsured patients (−$4,710; 95% CI, − $5,271 to −$4,148) and patients classified as other or no-charge (−$4,109; 95% CI, − $5,374 to −$2,843).

**Table 4:** Insurance-Stratified DID Estimates of the Association Between Medicaid Expansion and Total Hospital Charges Among Women Hospitalized with MDD, Comparing Maryland and New Jersey With Florida.

Insurance Type	State Comparison	Contrast ($)	Std. Error	t value	P value	95% CI
Uninsured	Maryland × Post-ACA	−4,709.65	286.50	−16.44	<0.001	−5,271.19 to −4,148.11
Medicare	Maryland × Post-ACA	−2,141.02	470.01	−4.56	<0.001	−3,062.24 to −1,219.81
Medicaid	Maryland × Post-ACA	−2,904.58	190.65	−15.24	<0.001	−3,278.25 to −2,530.92
Private insurance	Maryland × Post-ACA	−1,313.32	188.37	−6.97	<0.001	−1,682.52 to −944.13
Other / No charge	Maryland × Post-ACA	−4,108.81	645.63	−6.36	<0.001	−5,374.23 to −2,843.39
Uninsured	New Jersey × Post-ACA	−949.27	1,140.74	−0.83	0.405	−3,185.08 to 1,286.55
Medicare	New Jersey × Post-ACA	7,061.38	1,222.55	5.78	<0.001	4,665.22 to 9,457.53
Medicaid	New Jersey × Post-ACA	3,349.31	593.85	5.64	<0.001	2,185.38 to 4,513.25
Private insurance	New Jersey × Post-ACA	3,189.42	425.50	7.50	<0.001	2,355.46 to 4,023.39
Other / No charge	New Jersey × Post-ACA	2,451.32	1,166.50	2.10	0.036	165.02 to 4,737.62

Florida served as the non–Medicaid expansion reference state. Maryland implemented Medicaid expansion in 2014 and operates an all-payer hospital payment system, while New Jersey expanded Medicaid in 2014 without an all-payer model and was included as a sensitivity analysis. Estimates represent contrasts of predictive margins from multivariable linear regression models with robust standard errors, adjusted for age, race/ethnicity, median household income by ZIP code, primary expected payer, hypertension, diabetes, obesity, smoking status, and management of major depressive disorder (medical management, psychotherapy, and electroconvulsive therapy). Three-way interaction terms among Medicaid expansion status, ACA period, and race/ethnicity or insurance type were included to generate difference-in-difference-in-differences (DDD) estimates. The interaction term (Expansion × Post-ACA) represents the difference-in-differences estimator. Charges are reported in US dollars.

Reductions were also observed among Medicaid-covered (−$2,905; 95% CI, − $3,278 to −$2,531), Medicare-covered (−$2,141; 95% CI, − $3,062 to −$1,220), and privately insured patients (−$1,313; 95% CI, − $1,683 to −$944). In contrast, New Jersey experienced post-ACA increases in hospital charges among Medicare, Medicaid, privately insured, and other patients, while no statistically significant change was observed among uninsured patients. Together, these findings indicate that the association between Medicaid expansion and inpatient MDD hospital charges differs markedly by payer type and state payment environment.

## Discussion

In this multi-state analysis of women hospitalized with MDD, we examined whether Medicaid expansion was associated with changes in total inpatient hospital charges using data from the HCUP State Inpatient Databases. We found that hospital charges declined in Maryland following Medicaid expansion, while charges increased in New Jersey over the same period when compared with Florida, a non-expansion state. These findings indicate that the financial effects of Medicaid expansion on inpatient psychiatric care differ across states and are shaped by the broader health care policy environment.

A key feature that distinguishes Maryland from other expansion states is its All-Payer Hospital Payment System [32,33]. Under this model, hospital payment rates are set prospectively and applied uniformly across payers, including Medicare, Medicaid, and private insurers [34–37]. By limiting hospital price growth and reducing incentives for cost shifting between payer groups, the all-payer system promotes greater price stability and predictability in hospital financing [34–37]. Within this context, Medicaid expansion in Maryland may have translated more directly into lower hospital charges by reducing uncompensated care while simultaneously constraining hospitals’ ability to increase prices in response to changes in payer mix [38,39].

The consistency of charge reductions in Maryland across racial and ethnic groups and across insurance categories further supports this interpretation. The largest reductions were observed among uninsured and Medicaid-covered patients, populations most directly affected by coverage expansion and most vulnerable to financial hardship. These patterns suggest that expanding coverage within a regulated payment environment can yield meaningful financial benefits for patients requiring inpatient psychiatric care.

In contrast, New Jersey expanded Medicaid under a traditional hospital payment structure that allows greater flexibility in charge setting. In this setting, increased coverage did not correspond to lower hospital charges and was instead associated with higher inpatient costs for several payer groups. This divergence highlights that insurance expansion alone may be insufficient to moderate hospital charges in the absence of complementary payment or pricing reforms [39,40]. Taken together, these findings suggest that Medicaid expansion is most effective as a cost-moderating policy when paired with broader system-level mechanisms that promote price discipline.

### Policy and clinical implications

These findings have important implications for women’s health practice and policy. MDDH disproportionately affects women during critical life stages, including the reproductive and working years, when untreated psychiatric illness can adversely influence maternal health, chronic disease management, and overall healthcare utilization [41,42]. Reductions in inpatient charges associated with Medicaid expansion may improve financial access to essential psychiatric services and reduce barriers to acute mental health care. From a clinical perspective, policies that expand insurance coverage may facilitate earlier intervention and continuity of care, potentially mitigating the downstream consequences of severe depression. Collectively, these results highlight the intersection of mental health policy and women’s health and underscore the importance of coverage expansion in promoting equitable and affordable care for women.

### Study limitations

This study should be interpreted in light of several limitations. First, the analysis relied on administrative discharge data, which lack detailed clinical information such as symptom severity, treatment intensity, and outpatient follow-up. Although the case definition was restricted to ICD codes with established diagnostic correspondence to major depressive disorder, and non-specific codes such as F33.8 and F39 were intentionally excluded to minimize misclassification, reliance on administrative discharge codes may still introduce some degree of diagnostic imprecision, as these codes reflect billing-based documentation rather than standardized clinical assessment and may not fully capture the complexity of individual presentations. Second, although we used robust analytic approaches and conducted multiple sensitivity analyses, residual confounding related to unmeasured patient- or hospital-level factors cannot be ruled out. Third, while the extended pre-ACA (2007–2013) and post-ACA (2014–2020) periods were selected to capture stable trends before and after Medicaid expansion, this approach does not fully account for potential transitional effects during the early implementation years. Fourth, the comparison of a limited number of states may restrict generalizability to states with different demographic compositions, hospital market structures, or payment arrangements. Additionally, length of stay was not included in the primary models because it may lie on the causal pathway between Medicaid expansion and hospital charges. Adjusting for potential mediators could underestimate the total policy effect; however, the absence of this measure limits our ability to evaluate the extent to which changes in hospitalization duration contributed to observed charge patterns. TOTCHG may vary across states due to differences in billing practices, regulatory environments, and the inclusion or exclusion of specific charge components. Although HCUP employs standardized data-cleaning procedures to identify and correct implausible values, and we examined the distribution of charges for extreme values, residual variability may persist and could affect cross-state comparability. Furthermore, hospital charges reflect listed prices and should not be interpreted as actual costs, reimbursements, or patient financial burden. Charges are highly susceptible to variation in state pricing policies and institutional billing practices, and reductions in charges do not necessarily indicate equivalent reductions in costs to payers or patients. As such, observed charge differences may partly reflect changes in hospital pricing behavior rather than true differences in resource utilization. Hospital charges were analyzed in nominal dollars and were not adjusted for inflation over the 13-year study period (2007–2020), which may have contributed to observed increases in absolute charge levels across all states. Although the difference-in-differences design partially mitigates this concern by absorbing price trends common to all states into the time-period effects, residual inflation differences across states due to varying payment regulations or market structures cannot be excluded. Future studies should consider adjusting charges to constant dollars to facilitate more precise comparisons over time. Finally, Maryland operates under a unique All-Payer hospital payment model that regulates hospital pricing and may independently influence charge patterns. As a result, observed reductions in hospital charges may reflect the combined effects of Medicaid expansion and state-specific payment reforms. Although the inclusion of New Jersey—a state without an all-payer system—provides additional policy context, disentangling the independent contributions of coverage expansion and payment regulation remains challenging.

### Future directions

Future studies should examine the interaction between Medicaid expansion and hospital payment systems across a broader set of states to better understand the conditions under which expansion leads to lower inpatient costs. Incorporating patient-level measures of clinical severity, length of stay, and post-discharge outcomes would also help clarify the mechanisms underlying observed cost changes. In addition, evaluating outpatient mental health utilization and continuity of care may provide insight into whether lower inpatient charges reflect improved access to preventive and community-based services.

## Conclusion

In conclusion, Medicaid expansion in Maryland was associated with sustained reductions in inpatient hospital charges among women hospitalized with major depressive disorder, whereas similar reductions were not observed in New Jersey. These findings suggest that the financial impact of Medicaid expansion on inpatient psychiatric care depends in part on the surrounding payment and regulatory environment. Expansion policies implemented alongside mechanisms that limit hospital price growth, such as all-payer payment systems, may be particularly effective in moderating hospital charge growth, though whether these reductions translate into lower costs or reduced financial burden for patients warrants further investigation.

## Supporting information

S1 Table ICD-9 and ICD-10 codes used to define major depressive disorder. (DOCX)

S1 Fig Pre-expansion trends in mean log inpatient hospital charges (2009–2013). (DOCX)
