# Supplementary material for: Medicaid expansion and inpatient hospital charges among women with major depressive disorders
Source: PLoS One. 2026 Jun 25;21(6):e0335006. doi: 10.1371/journal.pone.0335006 (PMC13298965; doi:10.1371/journal.pone.0335006)
Supplement: S1 Table — (DOCX) [file pone.0335006.s001.docx]

# **Supplementary Table 1: ICD-9 and ICD-10 Codes Used to Define MDD**

| ICD-10 Code | ICD-10 Definition | ICD-9 Code | ICD-9 Definition |
| --- | --- | --- | --- |
| F32.0 | Mild depressive episode | 296.21 | Major depressive disorder, single episode - mild |
| F32.1 | Moderate depressive episode | 296.22 | Major depressive disorder, single episode - moderate |
| F32.2 | Severe depressive episode without psychotic symptoms | 296.23 | Major depressive disorder, single episode - severe without psychotic behavior |
| F32.3 | Severe depressive episode with psychotic symptoms | 296.24 | Major depressive disorder, single episode - severe with psychotic behavior |
| F32.4 | Depressive disorder, single episode, in partial remission | 296.25 | Major depressive disorder, single episode - in partial remission |
| F32.5 | Depressive disorder, single episode, in full remission | 296.26 | Major depressive disorder, single episode - in full remission |
| F32.8 | Other depressive episodes | 296.82 | Atypical depressive disorder |
| F32.9 | Depressive episode, unspecified | 296.20 / 311 | Major depressive disorder, single episode - unspecified / Depressive disorder NEC |
| F33.0 | Recurrent depressive disorder, current episode mild | 296.31 | Major depressive disorder, recurrent episode - mild |
| F33.1 | Recurrent depressive disorder, current episode moderate | 296.32 | Major depressive disorder, recurrent episode - moderate |
| F33.2 | Recurrent depressive disorder, current episode severe without psychotic symptoms | 296.33 | Major depressive disorder, recurrent episode - severe without psychotic behavior |
| F33.3 | Recurrent depressive disorder, current episode severe with psychotic symptoms | 296.34 | Major depressive disorder, recurrent episode - severe with psychotic behavior |
| F33.41 | Recurrent depressive disorder, in partial remission | 296.35 | Major depressive disorder, recurrent episode - in partial remission |
| F33.42 | Recurrent depressive disorder, in full remission | 296.36 | Major depressive disorder, recurrent episode - in full remission |
| F33.9 | Recurrent depressive disorder, unspecified | 296.30 | Major depressive disorder, recurrent episode - unspecified |
| F34.1 | Dysthymia | 300.4 | Dysthymic disorder |
