# Supplementary material for: Medicaid expansion and inpatient hospital charges among women with major depressive disorders
Source: PLoS One. 2026 Jun 25;21(6):e0335006. doi: 10.1371/journal.pone.0335006 (PMC13298965; doi:10.1371/journal.pone.0335006)
Supplement: S1 Fig — (DOCX) [file pone.0335006.s002.docx]

**Supplementary Figure S1. Pre-Expansion Trends in Mean Log Inpatient Hospital Charges, 2009–2013**

*This figure displays mean log inpatient hospital charges for Florida (non-expansion state), Maryland, and New Jersey (expansion states) during the pre–Affordable Care Act Medicaid expansion period (2009–2013). Visual inspection demonstrates similar trends in hospital charges across states prior to policy implementation in 2014, supporting the parallel trends assumption underlying the difference-in-differences design.*

**Parallel Trends Assessment**

To evaluate the parallel trends assumption underlying the difference-in-differences approach, we plotted mean log inpatient hospital charges across Florida, Maryland, and New Jersey during the pre-expansion period (2009–2013). Trends were largely parallel across states, with no evidence of substantial divergence prior to Medicaid expansion in 2014. These findings support the validity of the analytic design and strengthen confidence that post-expansion differences are unlikely to reflect pre-existing charge trajectories.
